# Sudden Cardiac Arrest During a Prolonged Liposuction and Lipofilling Procedure: A Case Report

**DOI:** 10.7759/cureus.25985

**Published:** 2022-06-16

**Authors:** Ahmed S Foula, Manal A Ahmed, Mohammed S Foula, Mohamed W Nassar

**Affiliations:** 1 Anesthesia, El Amrya General Hospital, Alexandria, EGY; 2 Anesthesia, Alexandria University Hospitals, Alexandria, EGY; 3 Surgery, King Fahd University Hospital, Imam Abdulrahman Bin Faisal University, Khobar, SAU; 4 Anesthesia, Faculty of Medicine, Helwan University, Helwan, EGY

**Keywords:** corticosteroids, acute respiratory distress syndrome, post cardiac arrest syndrome, lipofilling, liposuction, cardiac arrest, fat embolism syndrome

## Abstract

Liposuction is a popular cosmetic procedure. Recently, there has been an increase in the reported complications. Fat embolism syndrome (FES) is a rare life-threatening condition with challenging diagnosis. A young lady was admitted for liposuction and lipofilling procedure. After 180 minutes, cardiac arrest happened. She was revived after cardiopulmonary resuscitation. She was tachypneic, hypoxic, and feverish. Her chest x-rays were suggestive of acute respiratory distress syndrome. After exclusion of other differential diagnoses, she was diagnosed as post-arrest state on top of FES. Fortunately, she showed a gradual improvement, starting from the fourth day and was discharged to a regular ward on the sixth day.

Sudden cardiac arrest during liposuction is a dreadful complication that may occur in healthy persons due to FES. Its diagnosis depends on high index of clinical suspicion and use of special criteria and scoring systems. The management depends on conservative measures with/without steroids administration.

## Introduction

Liposuction is a popular cosmetic surgical procedure that can be performed using several surgical techniques, under local, regional, or general anesthesia. It was first performed in 1970s. It is considered as a relatively safe operation with a complication rate of 0.25% and an overall mortality rate of less than 0.002% [1]. However, with increasing numbers of the performed procedures, more complications have been increasingly reported. These complications range from minor (e.g., seroma, local anesthesia toxicity, asymmetry, and/or hyperpigmentation) to severe (e.g., visceral perforation, hemorrhage, venous thromboembolism, infection, necrotizing fasciitis, and fat embolism) [2]. Fat embolism syndrome (FES) represents one of the most serious complications after liposuction with or without lipofilling that may lead to significant morbidity and/or mortality [3].

FES is a rare, yet life-threatening condition that represents a systemic inflammatory response to circulating free fatty acids. Despite its rarity, FES may progress to a life-threatening situation and its mortality rate is reported as high as 15-36% [3,4]. Moreover, its diagnosis is challenging owing to its unspecific manifestations as well as unspecific lab works and imaging findings [5].

Herein, we present a case of cardiac arrest resulting from FES, during a prolonged liposuction procedure. We aimed to highlight its presentation during liposuction as well as its differential diagnoses and management dilemma.

## Case presentation

A 29-year-old female, who was not known to have any past medical illnesses, was electively admitted for liposuction of her abdomen, back, thighs, and upper arms as well as lipofilling of her breasts and buttocks. She was obese (class II obesity) with a body mass index of 38.5 kg/m^2^ (96 kg, 1.58 m). Her routine pre-operative blood works were unremarkable.

The patient was placed in a supine position with full basic monitoring according to the guidelines of the American Society of Anesthesia. Pneumatic compression was applied to her lower limbs. General anesthesia was induced and endotracheal tube was successfully inserted. Adequate hydration was ensured throughout the procedure.

The surgical procedure started with injection of tumescent fluid (containing adrenaline with concentration of 1/200,000 without local anesthesia) in the subcutaneous area followed by liposuction at the abdomen and both thighs. The procedure continued for 130 minutes in the supine position before turning the patient to the prone position for liposuction of the back and lipofilling of the buttocks. All connections were secured and good padding of the patient’s pressure points was checked.

After 180 minutes of the procedure and 6,500 mL of aspirated fluid, the capnogram readings showed an abrupt drop from 36 to 8 mmHg, before starting lipofilling. Sudden cardiac arrest was detected immediately with pulseless electrical activity rhythm. The patient was quickly reversed to the supine position. Two cycles of cardiopulmonary resuscitation (CPR) were required, without defibrillation, before return of spontaneous circulation. The patient had persistent hypotension after the CPR that required epinephrine infusion to maintain mean blood pressure (MBP) of 50-60 mmHg. The procedure was aborted and the patient was transferred to the surgical intensive care unit (SICU) for further management.

**Figure 1:**
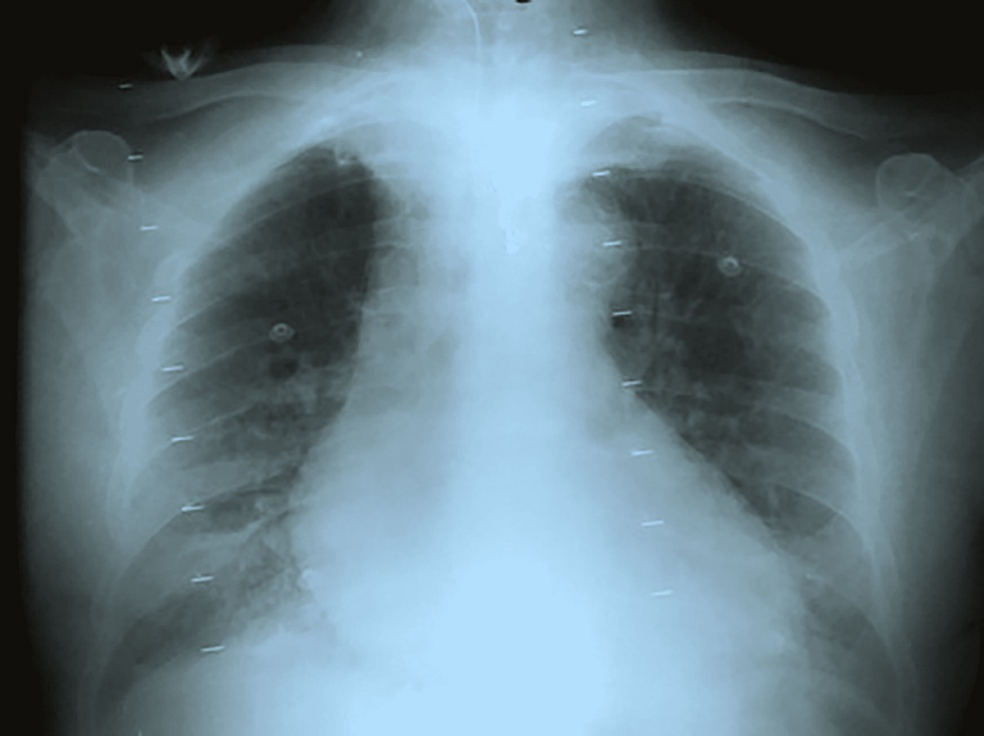
Chest plain radiography (portable) showing bilateral diffuse pulmonary opacities and cardiomegaly suggestive of ARDS. ARDS: acute respiratory distress syndrome

In the SICU, the vital signs showed respiratory rate of 45 per minute and temperature of 38.6°C. Her initial arterial blood gases showed severe hypoxia (PaO_2_ 62 mmHg on 100% oxygen). Moreover, there were copious frothy secretions coming from the endotracheal tube. She did not have any cutaneous manifestations. Upon admission to SICU, her immediate laboratory investigations revealed low hemoglobin and platelet levels as well as elevated total leukocyte count, c-reactive protein (CRP), serum lactate, and d-dimer levels. Her renal function tests and liver function tests were unremarkable (Table 1). Her initial chest x-rays showed bilateral diffuse alveolar opacifications suggestive of acute respiratory distress syndrome (ARDS) (Figure 1).

**Table 1:** The laboratory workup upon presentation. CRP: C-reactive protein; S. lactate: serum lactate

Blood test	Value	Reference range
Hemoglobin	9.3 g/dL	13.2-16.6 g/dL
White blood cells	12,200/mcL	4,000-11,000/mcL
Platelets	92,000/mL	150,000-450,000/mL
CRP	49.3 mg/L	<3.0 mg/L
S. lactate	2.9 mmol/L	0.5-1 mmol/L
D-dimer	6.8 mg/L	< 0.5 mg/L

Differential diagnoses at this point included severe hypovolemic shock leading to cardiac arrest, pulmonary macro-showers secondary to deep vein thrombosis (DVT), and FES. Hypovolemic shock was excluded by doppler assessment of the inferior vena cava that showed adequate filling with decreased compressibility (a picture consistent with post-arrest state), together with free sonographic assessment of the abdomen. Color Doppler assessment of venous system of both lower limbs failed to show any sign of DVT. Accordingly, pulmonary macro-showers were excluded. Her echocardiogram revealed a picture of global hypokinesia and ejection fraction (EF) of 38%. She was diagnosed as post-arrest state on top of FES.

Supportive treatment was continuous in the SICU in the form of noradrenaline infusion to maintain her MBP. Ventilatory support was done according to the ARDS protocol: low tidal volume, peak pressure control of 30 mmHg, no muscle relaxant, and permissive hypercapnia. Antibiotics, sedation, and analgesics were initiated according to the local hospital policy. Hydrocortisone 100 mg every six hours for five days was given as a steroid bolus therapy. On the second day, her PaO_2_ progressively dropped reaching 28 mmHg on 100% oxygen despite the continuous ventilatory support. Fever was persistent. Fortunately, the patient showed a gradual promising improvement, starting from the fourth day, in hypoxic index (PaO_2_ 101 mmHg on fraction of inspired oxygen FiO_2_ 0.7) that allowed gradual decrease of the inotropic support and weaning off from the mechanical ventilation.

On the sixth day, the general condition was stable and she was discharged to a regular ward. After three more days, she was discharged home in a good stable condition. She was followed up for three months in the cardiology clinic and her echocardiogram revealed improvement in form of good systolic functions (EF 58% with no resting wall motion abnormality).

## Discussion

Two different clinical entities are described in the literature: fat embolism and FES. Fat embolism is the presence of macroscopic fragments of fat in the blood circulation and has been frequently reported after liposuction and lipofilling procedures with an estimated incidence reaching up to 8.5% [6]. However, FES, which was first described after liposuction in 1986, differs as it represents a systemic multi-organ inflammatory response to the presence of fat embolism and may lead to multi-organ dysfunction [7]. In a recent systematic review including 104 articles and data from 3,583 patients and discussing the safety of large volume liposuction procedures (LVL), the authors defined LVL as removal of 5,000 mL of aspirate or more during a single procedure according to the definition of the American Society of Plastic Surgeons. However, they reported no cases of FES as a major complication after LVL [8].

The pathophysiology of FES includes endothelial injury by release of different inflammatory mediators and platelets aggregation [9]. Multiple theories have been postulated to explain occurrence of FES after liposuction. The mechanical theory suggested that fat droplets at the site of liposuction access the nearby injured vessels and travel in the systemic circulation till reaching the pulmonary vascular beds which may be blocked if the size of fat droplet is more than its diameter. Likewise, fat droplets may be deposited in any other organ such as brain [5,10]. The other theory depends on the biochemical effects of the free fatty acids which increase the systemic circulation owing to the effect of the catecholamine release in response to tissue trauma. The free fatty acids damage the pneumocytes and cause capillary endothelial damage thus increasing its permeability leading to chemical pneumonitis and development of ARDS. This theory is supported by the associated increased levels of cytokines and free radicals [5,11].

After liposuction procedures, FES severity is variable and may be subclinical [9]. The onset is usually gradual within the first 48 hours. The classical triad, described by Von Bergman, is present in only 0.5-2% of cases and includes respiratory, neurological, and cutaneous manifestations [4]. In general, the respiratory manifestations range from tachypnea to acute respiratory failure [9]. The neurological manifestations usually follow the respiratory manifestations and range from disorientation to coma. The skin manifestations include pathognomonic petechial rash in the upper body. Cardiac manifestations may present and vary from tachycardia to severe right-sided heart failure [5]. Sudden cardiac arrest is infrequently reported [3,5]. However, if FES occurs during the liposuction or lipofilling procedures under general anesthesia, the associated hypoxemia and tachypnea are usually masked by the mechanical ventilation support [4].

The laboratory abnormalities are also non-specific and include unexplained anemia, reduced hematocrit level, thrombocytopenia, hypocalcemia, and hyperlipasemia. Hypoxemia and hypocapnia are evident in the arterial blood gas analysis [5]. The presence of fat droplets in urine may help in diagnosis [6]. The gold standard imaging modality is contrast-enhanced computed tomography (CT) of the chest that may reveal diffuse infiltration with ground-glass pulmonary patches [9].

Despite it is difficult to early diagnose FES during liposuction procedures due to low specificity and sensitivity manifestations, early detection is crucial for proper management. Several diagnostic criteria have been developed. Gurd and Wilson’s criteria, described in 1970s, classified major and minor criteria. FES can be suggested by presence of two major or one major and four minor criteria (Table 2) [12]. In the fat embolism index, developed by Schonfeld et al., fat embolism is diagnosed with score equal to or more than five (Table 3) [13].

**Table 2:** Gurd and Wilson’s criteria for FES diagnosis. Two major or one major and four minor criteria suggest FES. FSE: fat embolism syndrome

Major criteria	Minor criteria
Axillary or subconjunctival petechiae	Tachycardia >110 beats/minute
Hypoxemia: PaO_2_ <60 mmHg, FiO_2_ = 0.4	Pyrexia >38.5℃
Pulmonary edema	Retinal fat or petechiae
Sudden drop in hemoglobin level >20%	Urinary fat globules or oligoanuria
Central nervous system depression disproportionate to hypoxemia	Sudden thrombocytopenia >50%
	Elevated erythrocyte sedimentation rate >71 mm/hour

**Table 3:** Schonfeld’s FES index. Five points or more is suggestive for FES. FSE: fat embolism syndrome

Findings	Points
Diffuse petechiae	5
Alveolar infiltrates	4
Hypoxemia (>70 mmHg)	3
Confusion	1
Fever (>38℃)	1
Heart rate >120 beats/minute	1
Respiratory rate >30 breaths/minute	1

Other differential diagnoses should be promptly ruled out before starting the management protocol including acute respiratory failure, venous thromboembolism, local anesthesia toxicity, and/or myocardial infarction [4]. The FES is usually a self-limited condition. Its management is controversial and mainly conservative consisting of adequate hydration, respiratory and hemodynamic support. Additionally, use of steroids has been suggested to decrease the associated inflammatory response and decrease the release of free fatty acids [6]. Yet, the ideal regimen of steroid administration is controversial [5]. Prevention of fat embolism during and after liposuction procedures is of utmost importance. Thus, some authors have recommended shorter surgical time, reducing the amount of suctioned fat, use of larger cannulas (>3 mm), gentle maneuvers, and availability of sufficient supporting resources and SICU backup [6].

In our case, the sudden decrease in her capnogram readings was the first alarming sign which was quickly followed by sudden cardiac arrest. After reviving, she had the classical cardinal signs of hypoxemia, tachypnea, and fever. she did not have any cutaneous manifestations. Her laboratory workup was only significant for new thrombocytopenia and elevated inflammatory markers. According to the previously mentioned diagnostic criteria, the patient developed two major and three minor criteria according to Gurd and Wilson’s FES criteria which is highly suggestive of FES. Moreover, according to Schonfeld’s FES index, the patient scored 10/16 points.

In the first couple of days, the patient was unstable to undergo contrast-enhanced chest CT. However, her chest radiography was suggestive of ARDS. Afterward, CT was not performed as FES was diagnosed using the diagnostic criteria, other differential diagnoses were excluded and, indeed, her condition was gradually improved. The associated low ejection fraction was attributed to post-cardiac arrest state and showed good improvement in the follow-up echocardiogram. Management was dependent on adequate ventilatory and hemodynamic support in addition to steroid administration. Continuous sedation in the SICU may be the cause of masking FES neurological manifestations. In addition, she had not developed any cutaneous manifestations. Fortunately, her condition gradually improved and she had no complaints during her follow-up visits to cardiology and plastic surgery outpatient clinic.

## Conclusions

Sudden cardiac arrest during liposuction and lipofilling procedure is a dreadful complication that may occur in healthy persons due to FES. Prevention of FES is important and several technical tips have been recommended as shorter surgical time, less amount of suctioned fat, and use of larger cannulas. Its diagnosis depends on high index of clinical suspicion and use of special criteria and scoring systems. Other differential diagnoses should be excluded as the management is different. The management depends on conservative measures with/without steroids administration. The outcome depends on the early diagnosis and proper management protocol.
